# P87 - A national multicenter registration of treatment with omalizumab in Danish asthmatic children

**DOI:** 10.1186/2045-7022-4-S1-P142

**Published:** 2014-02-28

**Authors:** Katrine B Spangenberg, Inger Merete Jørgensen, Susanne Halken, Lone Agertoft, Frederik Buchwald, Sune Rubak, Margrethe Friberg, Henrik Fomsgaard Kjær, Thomas Petersen Houmann, Thomas Kongstad

**Affiliations:** 1 Pediatric Department, Hillerød University Hospital, Denmark

## Background

In Denmark, Omalizumab is approved for treating children with severe persistent allergic asthma older than 6 years of age. No systematic registration of the efficacy in the Danish child population according to asthma symptoms or the efficacy on co-morbid allergic symptoms exists. A broad panel of outcome measures is necessary to evaluate the efficacy of Omalizumab treatment.

## Aim

To provide a standardized systematic registration in order to create a database enrolling children with severe allergic asthma treated with Omalizumab.

## Method and study design

A national multicenter registration and follow-up study based on children with clinical persistent severe allergic asthma including both retrospective and prospective registration.

### Inclusion criteria

Children, 6-18 years of age, with severe persistent allergic asthma according to GINA treated with Omalizumab.

### Outcome registration

A broad panel of outcome measures is scheduled at baseline and during treatment:

### Outcome

• Asthma exacerbations

• Hospitalizations

• Medication

• Lung function

• ACT score

• Peak-flow

• FeNO

• Mannitol/Methacholin test

• Skin prick-test

• Quality of life score. (Juniper)

• Rhinitis score, R-RTSS

• SCORAD score

The monitoring plan and follow-up programme will be presented in a flowchart.

## Conclusion

We believe that a national systematic registration will increase knowledge regarding efficacy of Omalizumab treatment in this rare group of severe allergic asthmatic children.

